# Notes and News

**Published:** 1889-07-20

**Authors:** 


### Notes and News.

Collected and Communicated.

Amongst the lectures to be delivered at Blackpool in connection with the Home Reading Union are a course by Messrs. Kimmins and Laurence on "The Chemistry of Life and Health," one by the Master of Downing on "Our Thinking Apparatus," and one by Dr. Courtney S. Kenney on "English Charity and English Charities." The last should break new ground, and prove of unusual interest.

Refreshments provided by the Native Guano Company, and a visit to the new Kingston and Surbiton Sewage Works, doesn't sound exactly cheerful, and yet the members of the Society of Chemical Industry thoroughly enjoyed their annual outing. They visited some dozen different establishments and saw the process of refining sugar, of tanning by electricity, and the Brush electric light.

The annual commemoration of the New York College of Physicians and Surgeons took place on June 13 in the college building, Tenth-avenue and Fifty-ninth-street. The graduating class numbered 166. Bishop Potter, as chaplain, Prof. J. G. Curtis, Acting-President Drisler, and the vice-president, Dr. Thomas M. Markoe, took part in the exercises, the latter making an address to the class. The alumni prize of 500 dollars, offered by Dr. Cartwright, and open to any alumnus of the college, for an essay on original work, went to Dr. H. A. Hare and Dr. Edward Martin, of the University of Pennsylvania.

Amongst the chief prize-takers at Guy's last week were: The Treasurer's Gold Medals for Clinical Medicine and Clinical Surgery, Mr. Alfred Parkin, Hightown, Yorkshire; the Gurney Hoare Prize for Clinical Study, Mr. Albert E. Norburn, Stoke Newington ; the Beaney Prize for Pathology, Mr. R. D. Mothersole, Colchester; the Sands Cox Scholarship and the Michael Harris Prize for Anatomy, Mr. A. T. Rake, Fordingbridge. During the afternoon the wards and other parts of the hospital, so far as was practicable, were thrown open to the visitors, while in the grounds, which were prettily decorated, a selection of music was given by the band of the M Division of Police.

The proceeds of the Hospital Saturday Fund were counted on Monday night by a band of volunteer bank clerks. There were over 2,000 boxes, and owing to the fine afternoon they were fairly filled. One or two nurses were pressed into the service of the fund this year, and their uniforms attracted attention, and gave them time to beg for donations. The collectors were much more numerous in the City than in more fashionable neighbourhoods ; they were more persistent and more successful.

St. Gabriel's, Canning Town, issues a tiny leaflet monthly from which we learn that from October, 1888, to June, 1889, dinners to the number of 11,135 were provided for children, and to the number of 135 for sick persons. Also, that £6 16s. 10d. was collected on Hospital Sunday, being the result, apparently, of much labour in a poor neighbourhood. In the church the offertory was only £1 8s. 11d., but ten collectors went forth into the streets, including the Rev. C. H. Clarke, who gathered together 12s. 6d.

The medical world has gone in violently for dissipation lately, and dinners and *soirées* have taken place nightly. Mrs. Critchett was at home in Harley-street, last week, to over a hundred guests, including Lady Mackenzie, Dr. Robson Roose, and others. Then Dr. Robson Roose gave a dinner to meet General Boulanger; and Mrs. Lennox Browne gave a *soirée* at which Sir Morell Mackenzie was the chief guest. How our great consulting physicians find time to attend to patients, to attend numerous meetings on professional subjects, and to yet enjoy social frivolities, it is hard to say.

The Marchioness of Waterford kindly permitted the annual meeting of the Mothers' Lying-in Home, Shadwell, to be held at her residence last week. The Marquis of Waterford presided, and there were a large number of ladies present. Princess Frederica of Hanover, who had announced her intention to attend the meeting, was unavoidably prevented from doing so. From the annual report it appeared that since the opening of the home on December 6, 1884, to April 30, 545 patients had been registered for admission; 309 had been admitted and discharged well, and 59 were attended at their own homes. During the past year 125 patients were treated with the most satisfactory results.

An amusing incident came to our notice at the recent reopening of the Phœnix Mills, at Dartford, by Messrs. Burroughs, Wellcome, and Co., of Snow-hill, London. Floral and other decorations were prepared, and everything that could possibly add to the brightness and comfort of visitors was done. On the morning of the opening day, Dr. Saxton, late of Philadelphia, U.S.A., who is now attached to the staff of the firm, remarked to the manager of the works that it was a pity that the newly-planted rose-trees were not contributing to the gaiety of the scene. The manager acquiesced, with the additional remark that "it could not be helped." "Oh, yes it can—I'll make 'em bloom," said the doctor as he walked away. In the afternoon the rose-trees were the admiration of everyone, and the Vicar of Dartford, who took the chair, in notifying the wonderful transformation which had come over the mills since the firm had taken to them, illustrated his remarks by referring to the sudden blooming of the rose-trees. The doctor on leaving the manager in the morning went and bought a basketful of the finest blooms that Dartford nurserymen could supply, and with the help of some very fine wire the trees were made to bloom for the occasion, as, perhaps, they would not have done, except in the presence of a 'cute American. In order to prevent discovery by visitors plucking the roses, a supply of cut roses was distributed liberally amongst the ladies.

The following vacancies are announced this week, the amount of the salary in each case being given after the name of the institution :

**Resident Medical Officers.**

Deaconesses Institution, Tottenham. House Surgeon.—£25.
West Cumberland Infirmary. House Surgeon.—£120, apartments, &c.
Rubery Hill Asylum. Clinical Assistant.—Board, &c.
Hockley Provident Dispensary. Medical Officer.—£250, residence, &c.
Northumberland County Asylum. Clinical Assistants.—Board, &c.
Royal Berks Hospital. Assistant House Surgeon.—£40, board, &c.
Birmingham General Hospital. Assistant House Surgeon.—Board, &c.

**Non-Resident Medical Officers.**

City of London Hospital, Victoria Park. Assistant Physician.
Royal College of Physicians. Imlroy Lecturer.
Hospital for Women, Soho-square. Clinical Assistants.
Wirral Hospital. Honorary Acting Medical Officer.

The Shah has exhibited no great interest in our medical charities, though the hospitals have not been behindhand in decorating in his honour. Still, His Highness the Amin-es-Sultan, First Minister of the Persian Court, has visited St. George's Hospital. He was conducted round the wards, and on leaving expressed his great gratification with all he had seen. He subsequently sent a donation of one hundred Russian half-imperials to the funds of the hospital.

Much correspondence is going on in the local press just now anent the Coventry Provident Dispensary, which, it is said, is abused rather than used. That 20,000 persons obtain medical advice and medicine (at one institution) for one penny per week, and twopence halfpenny in the case of married persons, is a scandal and a disgrace to the city, because 15,000 are in a position to pay for both, and it is a gross injustice to medical men outside the dispensary and also to chemists and druggists. But then the figures are not ours ; but if they are correct, it certainly seems desirable to alter the rules.

The Nizam of Hyderabad issues a practical challenge to the *Lancet*, so sure is he of the truth of the theory regarding chloroform made public by Dr. Lawrie. A few months ago we gave a short note on the result of the Hyderabad Commission on chloroform, and on the experiments there performed. The *Lancet* need not have been so chary of acknowledging the possibility that chloroform does not affect the heart until after the breathing has stopped, for some of our most noted anæsthetists never dream of examining the heart or feeling the pulse, but trust simply to skill in administration. An eminent oculist, who has some twenty patients under chloroform and ether daily, has never had a death due to chloroform yet, and he never examines the patient's heart beforehand.

The annual meeting of the committee and subscribers of the Central London Throat and Ear Hospital, of which the Archbishop of Canterbury is president, was held in the board-room on Friday afternoon, Captain Hutton, chairman and treasurer, presiding. The secretary, Mr. R. Kershaw, read the report, which showed that 6,257 new out-patients had been treated in the past year, involving 31,589 separate attendances. The number of in-patients had been 230. The income had been £2,800, which included a legacy of £500. The current expenses had been £2,169, and beyond this the sum of £750 had been paid off the loan of £1,000 from the bankers. The liabilities at the present time were £973, and with this exception the charity was free of debt. An earnest appeal is made for funds to defray this indebtedness.

At the Suffolk Assizes, held at Bury St. Edmunds, before the Lord Chief Justice, Mr. James Bond, farmer, of Bushall, near Stowmarket, sued Mr. Henry Payne Leech, surgeon, of Woolpit, to recover damages alleged to have arisen through unskilful and negligent surgical treatment. More than three years ago plaintiff was riding a horse which reared and fell upon him, breaking his right thigh. Plaintiff sent for defendant, whose son, who acted as assistant to his father, attended. The plaintiff maintained that he was unskilfully treated, with the result that he was still a cripple. The jury awarded plaintiff £290 damages.

A most interesting meeting was held last week by the Association for the Oral Instruction of the Deaf and Dumb. Mr. Van Praagh gave an illustration of the system, and his pupils understood and answered him in a marvellous manner, simply reading his meaning by the movements of his lips. During last year thirty-two boys and nineteen girls have attended the school. Students are also trained in the work of teaching. Some of those trained at the college are now employed by the London School Board ; one has gone to China, and others are engaged in private tuition. The report of the Royal Commission on the Blind, the Deaf, and the Dumb is now before the House of Commons, and calls attention to this system of oral instruction.

The festival dinner in aid of the funds of the Metropolitan Hospital was held last week, the Rev. William Rogers, rector of Bishopsgate, presiding. Besides the claims for public support which arise from the fact that this institution is situated in the very heart of one of the poorest and most crowded parts of the East End of London, the Metropolitan Hospital possesses yet another remarkable feature. It is conducted on provident principles. For over eighteen months the committee have encouraged persons living within a mile radius, who are really too poor to pay the ordinary fees of medical men, to provide a small, regular, monthly sum, in health as well as in sickness, this entitling them to all the relief which a general hospital can afford, in event of its being required. In order to prevent abuse of the system by those who can well afford to pay a doctor, a wage limit is fixed, and only those are permitted to join the provident department whose average wage earnings do not exceed that limit. That the poor residents in the neighbourhood of the Metropolitan Hospital appreciate this system is clear from the fact that in the 18 months referred to 5,654 membership books have been issued, representing more than 11,000 lives. Although the hospital has accommodation for 160 in-patients (including the Hebrew wards, which will contain 40 beds in a separate block, with separate kitchen, cook, etc.), only 51 beds have been utilised, owing to a want of funds. The result of the festival was an addition of £2,014 8s. 9d. to the funds, which is satisfactory. Amongst the speakers was Sir Edmund Hay Currie, to whose devotion and labour the People's Palace and other London charitable and social institutions owe so much. Sir Edmund, after living, as he said, for thirty-five years in the East of London, believes that all poor residents appreciate the provident system, which gives them a right to relief and renders them independent of charity. He is, therefore, a warm upholder of the system, and believes that all newly established hospitals should adopt the provident principle. Sir Edmund expressed the belief that the time had come when hospitals should be moved to where they are most wanted, and that their management generally should be looked into. He eulogised the unselfish labours of medical men, and denounced the numerous "quacks" which frequent East London. Mr. Charles H. Byers, the secretary, was also referred to in terms of the highest praise.

